# 

**Published:** 1910-04-02

**Authors:** 


The Governors of the Royal Edinburgh Hospital for Sick Children recently decided that the appointment of a lady visitor would be of great value to the hospital. Her duties would be to keep in touch with patients who had received advice and medicine at the out-patient department, and to ascertain that such instructions as had been given were being carried out. She would also be able to give to the mothers of patients hints in regard to the details of the treatment prescribed, and thus secure more efficient care in the case of children nursed in their own homes. They have accordingly appointed as hospital visitor Mrs. Mary C. Gunn, who has had large experience in visiting among the poor in Glasgow under the schemes of the Charity Organisation Society.

THE HOSPITAL. April 2, 1910.

*Name* ..........................................

*Address* ..........................................

..........................................

This Coupon must accompany manuscript or contributions intended for The Hospital.

### THE MEDICAL LIBRARY.

Post Free.

"Medical History: From the Earliest Times." By E. T. Withington, M.A., M.B. Oxon ... 12s. 6d.

"The Consumptive Working Man. What Can the Sanatoria Do for Him?" By Noel D. Bardswell, M.D. ... ... ... ... ... ... 10s. 10d.

"Photomicrography." By Edmund Spitta, M.R.C.S. With 41 Half-tone Reproductions from original negatives and other Illustrations ... ... 12s. 0d.

Of all booksellers or of The Scientific Press, Limited, 28 and 29 Southampton Street, Strand, London, W.C.

### Appointment.

Evans E. Laming, M.A., M.D., B.C. (Cantab), F.R.C.S. (Eng.), has been appointed surgeon to the Surgical Aid Society, Salisbury Square, E.C.

### THE BOLINGBROKE HOSPITAL AND THE WANDSWORTH BOROUGH COUNCIL.

At the last meeting of the Wandsworth Borough Council it was decided to lodge an appeal against the recent decision of Mr. Justice Eve, which awards the Weir bequest to the Bolingbroke Hospital. The Rev. J. H. Anderson, in moving the adoption of the report, said the judgment ought obviously to be appealed against, otherwise Councillors would find it very difficult to get their constituents to understand why a gift yielding £3,000 per annum specifically made to Streatham should have been handed over to Battersea. The Judge's remarks were in themselves an invitation to go to the Appeal Court. Mr. Hewett seconded. Mr. Lidiard said if they proceeded further, and were defeated, members would probably have to pay the costs out of their own pockets. Mr. Lorden, L.C.C., said he would not mind paying £10 to safeguard the right of every man to have his posthumous wishes carried out. If he chose to leave his money to a home for lost toads he did not see that it would be anyone's business to interfere. The Town Clerk, in reply to a question, said he was of opinion that the Council could incur the costs. Dr. Robinson and other members maintained that the wisest interpretation of Mr. Weir's wishes would be to apply the money to the Bolingbroke Hospital, which could confer more benefit on Streatham than any local institution. On a division it was decided to take steps to appeal.

### Gifts and Bequests.

The Royal Waterloo Hospital for Women and Children has received a further donation of £100 from "A. B."

The Surgical Aid Society acknowledges the receipt of £100 from the estate of the late Mr. J. Mackrell, and £10 10s. each from the Dyers' Company and from Harrods' Benevolent Society.

Mr. Moritz Von Halle, of Rossefield, Park Drive, Heaton, Bradford, stuff merchant, of the firm of Messrs. L. N. Hardy and Co., who died on January 30, aged 69, left £625 in charitable bequests, including £300 to the Bradford Royal Infirmary.

The Goldsmiths' Company has awarded grants of £250 each to the Mount Vernon Hospital for Consumption and the Westminster Hospital.

Mr. John Cory, coalowner, of The Duffryn, near Cardiff, left £5,000 to the Glamorgan and Monmouthshire Infirmary, Cardiff.

The directors of the Bank of England have sent a donation of £105 to the Royal Hospital for Diseases of the Chest, City Road, E.C.

The legacies and donations of £100 and upwards to the Edinburgh Royal Infirmary during the past year amounted to £20,665 3s. 4d., which included £820 specially destined to permanent capital.

The Hospital for Consumption and Diseases of the Chest, Brompton, has received a donation of £500 from the Goldsmiths' Company in aid of the Sanatorium and Convalescent Home at Frimley.

The Hon. Secretaries of King Edward's Hospital Fund for London have received at the Bank of England from the executors of the late Miss Allen Morrison the sum of £2,000, being the amount of a legacy (free of duty) bequeathed by her to the Fund.

A meeting of railway-men was held last week at Percy Main for the purpose of allocating the half-yearly collections. It was decided that £10 be given to Newcastle Infirmary, £3 to Tynemouth Infirmary, £1 to the Eye Infirmary, £1 to the Ear Infirmary, £1 to the Fleming Memorial Hospital, and £1 to the Chest Hospital.

### Jottings.

The managers of the Edinburgh Royal Infirmary have named a bed in a male surgical ward in commemoration of the bequest by Miss Moncrieff Arnott, of Chapel.

Lady Salisbury will visit the Royal Hospital for Incurables, Putney Heath, on Tuesday, June 7, at 2.30, when she will open a three-days' sale of work. The bulk of the needlework, etc., which will be offered for sale is the result of many months' industry on the part of in-patients.

The Governors of St. Bartholomew's Hospital will meet the Governors of the other Royal Hospitals at Christ Church, Newgate Street, on Wednesday, April 6, at a quarter before three o'clock in the afternoon, to hear the annual hospital sermon.

The annual general meeting of subscribers to the Hospital for Invalid Gentlewomen will be held on the new premises at 19 Lisson Grove, N.W., on Monday, April 4, at 3 p.m. Earl Waldegrave in the chair.

The annual festival dinner of the Royal Hospital for Incurables will be held at the Grocers' Hall, Princes Street, E.C., on Wednesday, May 11, 1910, at 6.30 p.m. for 7 p.m. precisely, Sir Horace Brooks Marshall, M.A., LL.D. (Alderman of the City of London), in the chair. Ladies are cordially invited to be present at the dinner. Gentlemen who are willing to act as stewards should kindly communicate with the Secretary.

The working-men's committee has presented Grimsby Hospital with a light department equipped with every apparatus for the light cure of diseases and $x$-ray work. The installation, one of the finest in the provinces, is the gift of the charity gala committee, which has already presented the institution with two new wards and large sums of money. In making the presentation the chairman said that the committee's next gift will take the form of a tube of radium.

At the Benenden Sanatorium, Kent—the first erected, under the auspices of the Hospital Saturday Fund, by the National Association for the Establishment and Maintenance of Sanatoria for Workers Suffering from Tuberculosis—there were 229 patients who were treated and left the institution during last year. A feature of the year's work was the provision of accommodation for eleven more patients by the erection of a new pavilion.

**For Hospitals and Institutions.**

| | |
|---|---|
| Analysis Ledger. 3 vols. (Uniform with the new Index of Classification adopted by the King's Hospital Fund) | £1 12s. 6d. |
| Cash Book | 12s. 9d. |
| Cash Analysis and Receipt Book | 16s. 6d. |
| Secretary's Petty Cash Book | 13s. 0d. |
| List or Register of Annual Subscribers | £1 0s. 0d. |
| Linen Register | 7s. 6d. |

And many other Ledgers necessary in the office of large and small institutions.

Of all booksellers or of The Scientific Press, Limited, 28 and 29 Southampton Street, Strand, London, W.C.

